# Comparative Transcriptome Profiling Unfolds a Complex Defense and Secondary Metabolite Networks Imparting *Corynespora cassiicola* Resistance in Soybean (*Glycine max* (L.) Merrill)

**DOI:** 10.3390/ijms241310563

**Published:** 2023-06-23

**Authors:** Sejal Patel, Jinesh Patel, Katherine Silliman, Nathan Hall, Kira Bowen, Jenny Koebernick

**Affiliations:** 1 Department of Crop, Soil and Environmental Sciences, Auburn University, Auburn, AL 36849, USA; 2 School of Fisheries, Aquaculture and Aquatic Sciences, Auburn University, Auburn, AL 36849, USA; 3 Department of Entomology and Plant Pathology, Auburn University, Auburn, AL 36849, USA

**Keywords:** target spot, RNA-Seq, soybean, *C. cassiicola*, differentially expressed genes, resistance, defense response

## Abstract

Target spot is caused by *Corynespora cassiicola*, which heavily affects soybean production areas that are hot and humid. Resistant soybean genotypes have been identified; however, the molecular mechanisms governing resistance to infection are unknown. Comparative transcriptomic profiling using two known resistant genotypes and two susceptible genotypes was performed under infected and control conditions to understand the regulatory network operating between soybean and *C. cassiicola*. RNA-Seq analysis identified a total of 2571 differentially expressed genes (DEGs) which were shared by all four genotypes. These DEGs are related to secondary metabolites, immune response, defense response, phenylpropanoid, and flavonoid/isoflavonoid pathways in all four genotypes after *C. cassiicola* infection. In the two resistant genotypes, additional upregulated DEGs were identified affiliated with the defense network: flavonoids, jasmonic acid, salicylic acid, and brassinosteroids. Further analysis led to the identification of differentially expressed transcription factors, immune receptors, and defense genes with a leucine-rich repeat domain, dirigent proteins, and cysteine (C)-rich receptor-like kinases. These results will provide insight into molecular mechanisms of soybean resistance to *C. cassiicola* infection and valuable resources to potentially pyramid quantitative resistance loci for improving soybean germplasm.

## 1. Introduction

Soybean (*Glycine max* (L.) Merrill) is widely consumed in many forms, contributing 59% of the world’s edible oil production and supplying 31–40% of high-quality protein, making it a good staple food source for human and animal consumption. In 2021, 128 million ha of soybean was produced with yields of 364 million metric tons [1]. Soybean production typically does not achieve its full yield potential due to biotic stresses [2].

Growers in the southern US soybean industry are facing a serious challenge posed by the plant pathogenic fungus, *Corynespora cassiicola* ((Berk. & M.A. Curtis) C.T. Wei). This fungus causes target spot and thrives in warm and moist environments, leading to estimated yield losses of 18–32% [3,4]. Symptoms of infection by *C. cassiicola* are necrotic spots with alternating concentric rings of light and dark brown bands, usually encircled by a yellow halo on the foliage. In addition, lesions on stems, pods, and seeds, and premature leaf senescence could occur in severe cases [3,5]. The present method for controlling target spot relies on fungicide applications. The immense use of fungicides can lead to the development of resistance in *C. cassiicola* isolates and reduce the effectiveness of fungicides [6,7,8]. A more eco-friendly and sustainable approach is to use resistant cultivars.

*Corynespora cassiicola* resistance exists in soybean genotypes [9]; however, the genes and mechanisms of resistance are unknown. Investigating disease resistance mechanisms relies on the identification of genomic regions, genes, and gene networks associated with defense responses triggered by the host upon infection with a pathogen [10]. RNA sequencing (RNA-Seq) is an advanced and effective technology used for studying gene expression at the whole genome level which can detect novel transcripts, splice junctions, facilitates DEG analysis, and allow functional gene mining [11,12]. The enhancement of high-throughput sequencing (HTS) technology and the availability of comprehensive soybean genome sequences have allowed the full-scale examination of the transcriptomic response to disease [13]. Soybean RNA-Seq studies have provided an opportunity to gain in-depth knowledge of plant–pathogen interactions by identifying responsive genes and pathways for disease resistance such as to soybean cyst nematodes [14,15], Phytophthora root and stem rot [16], Fusarium root rot [17], bacterial leaf pustules [18], *Soybean mosaic virus* [19], downy mildew [20], and brown stem rot [21].

*Corynespora cassiicola* is a devastating pathogen in crops such as rubber tree (*Hevea brasiliensis*), cotton (*Gossypium hirsutum* L.), and cucumber (*Cucumis sativus* L.) [22,23,24]. RNA-Seq studies in cucumber and rubber, after infection by *C. cassiicola*, have found differential gene expression associated with Ca^2+^ signaling pathways, pathways targeting salicylic acid (SA), ethylene (ET), and phenylpropanoid biosynthesis [25,26,27,28]. Such studies have aided in the identification of miRNAs, genes, and gene variations that are critical for understanding the genetic basis and marker development for disease resistance. In soybean, histochemical characterization and biochemical assays in control and infected tissues of the plant suggest a major role of total soluble phenolics (TSP) and lignin-thioglycolic acid (LTGA) derivatives in controlling the spread of *C. cassiicola* in leaf tissue [29,30]. However, to date, there have been no transcriptome studies for the *C. cassiicola* interaction with soybean to validate such claims. Two genotypes, Bedford and Council, have some level of resistance as low levels of disease developed on each after inoculation with *C. cassiicola*; two other genotypes, Henderson and Pembina, are documented as susceptible [9]. The objective of this study is to conduct a comparative transcriptomic analysis to determine the differentially expressed genes (DEGs) between non-inoculated control and post-*C. cassiicola* infection at 24 and 48 h post-infection. These DEGs will be further evaluated for Gene Ontology (GO) and Kyoto Encyclopedia of Genes and Genomes (KEGG) pathways. This will provide insight into genes and molecular mechanisms that underlie soybean resistance to *C. cassiicola* infection.

## 2. Results

### 2.1. RNA Sequencing Data Analysis and DEGs in Response to C. cassiicola

**Figure 1:**
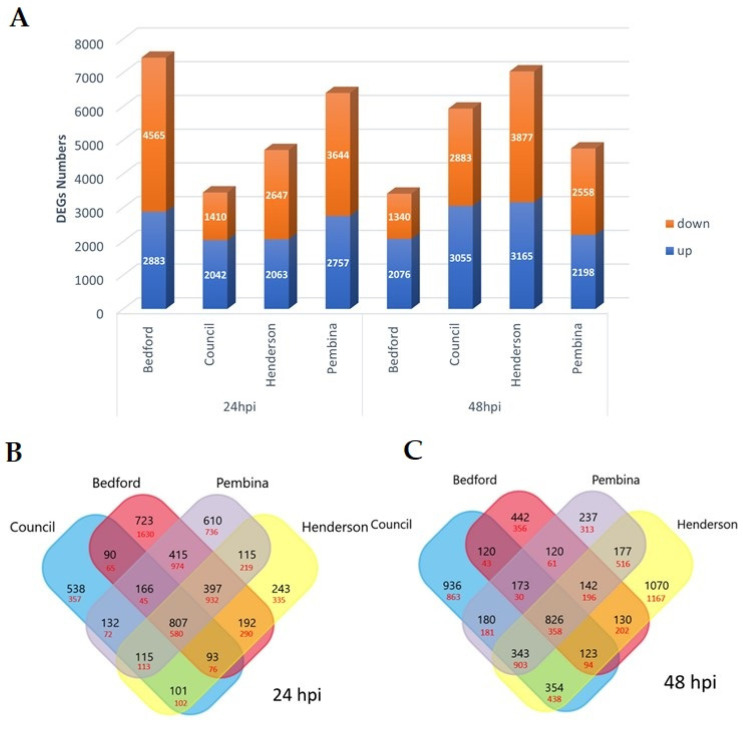
Differntially expressed genes (DEGs) retrieved from all four genotypes at 24 hpi and 48 hpi time intervals compared to non-inoculated control. (**A**) Total numbers of DEGs (upregulated and downregulated) at each time point. Venn diagram illustrating comparison of DEGs (**B**) at 24 hpi and (**C**) at 48 hpi, among all four genotypes, both resistant (Bedford and Council) and susceptible (Henderson and Pembina). Black-colored numbers represent upregulated genes, and red-colored numbers represent downregulated genes.

For RNA sequencing, raw reads ranged from 22,838,228 to 33,485,280 per sample, with an average GC content of 44–45%. After quality control, the adapter and low-quality reads were discarded from the data. The clean data ranged from 22,381,404 to 33,067,807 reads per sample (Table 1). A total of 88–91% of the sequence reads were successfully mapped to the soybean reference genome. A total of 11,263 DEGs were identified at 24 hpi, including 1387 DEGs that were common in all four genotypes. Moreover, a total of 11,094 DEGs were determined at 48 hpi, including 1184 DEGs that were common in all four genotypes (Figure 1). The number of up- and downregulated genes differed between the four genotypes. Specifically, Bedford and Pembina had more differentially expressed genes at 24 hpi, whereas Council and Henderson had more at 48 hpi (Figure 1).

A total of 5172 genes at 24 hpi and 5384 genes at 48 hpi were uniquely differentially expressed in one of the four genotypes after *C. cassiicola* infection. In the resistant genotypes, 3403 and 2760 genes were differentially expressed at 24 hpi and 48 hpi, respectively, that were not expressed in susceptible genotypes. Unique DEGs in Council were 895 at 24 hpi and 1799 at 48 hpi; for Bedford, there were 2353 at 24 hpi, and 798 at 48 hpi. A total of 155 and 163 DEGs were shared in both resistant genotypes at 24 hpi and 48 hpi, respectively (Figure 1B,C).

A total of 334 and 693 DEGs were shared between susceptible genotypes at 24 hpi and 48 hpi, respectively. The unique DEGs in Henderson were 578 at 24 hpi and 2237 at 48 hpi; in Pembina, there were 1346 DEGs at 24 hpi and 550 DEGs at 48 hpi (Figure 1B,C).

### 2.2. Gene Ontology (GO) Enrichment Analysis of DEGs

**Figure 2:**
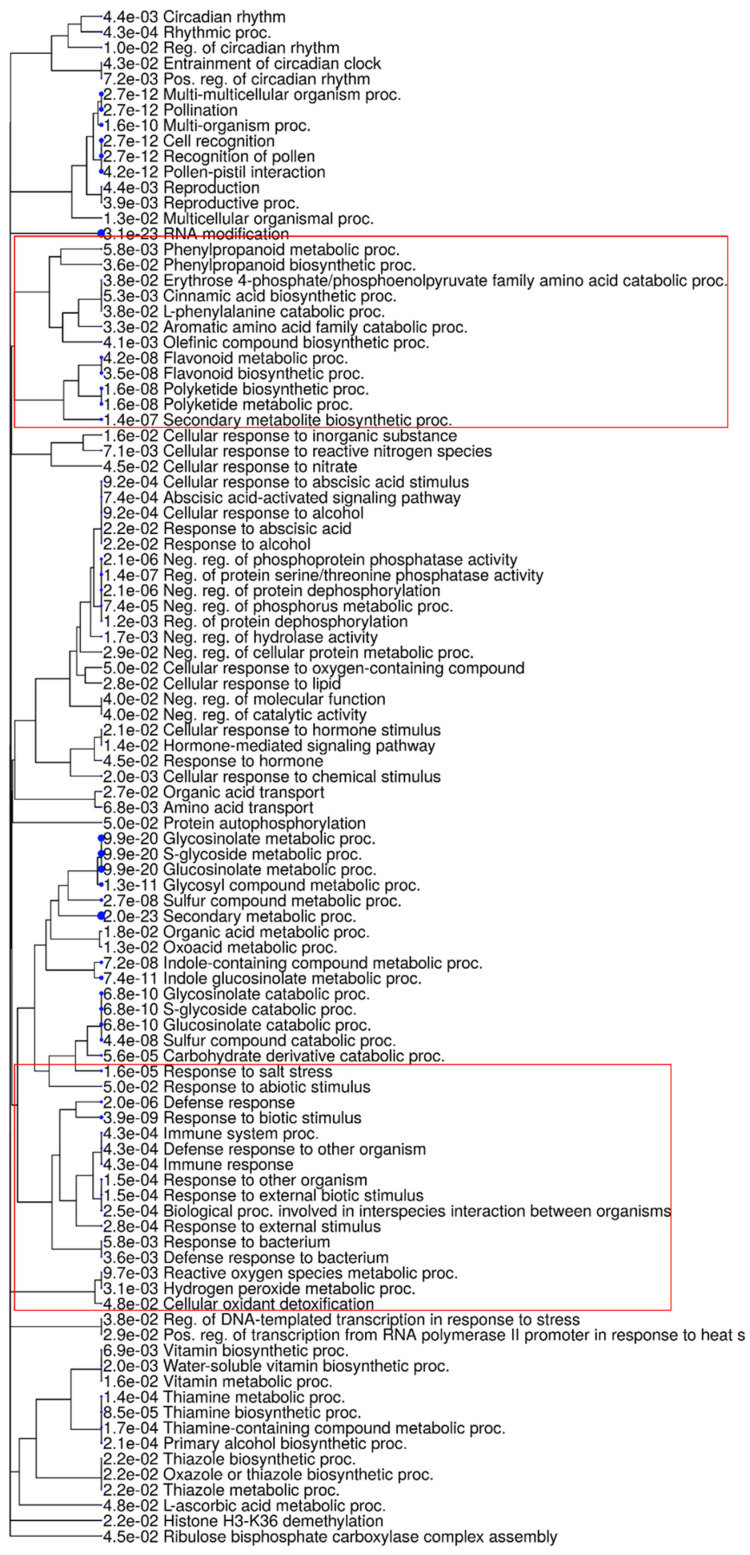
A hierarchical clustering tree of enriched biological processes. This clustering summarizes the correlation between significant pathways and pathways are clustered together if they share any common genes. The Gene Ontology (GO) terms in the red box are some biosynthesis pathways and responses activated after a pathogen attack.

The results of GO enrichment for the DEGs common in all four genotypes after *C. cassiicola* infection, regardless of time after inoculation, revealed 150 GO terms and 139 GO terms for upregulated and downregulated DEGs, respectively. The enriched GO terms in upregulated biological processes were the secondary metabolic process, RNA modification, the glucosinolate metabolic process, cell recognition, response to biotic stimulus, the flavonoid biosynthetic process, regulation of protein serine/threonine phosphatase activity, the defense response, and the immune response, indicating the importance of the activation of a defense-related network in response to *C. cassiicola* infection. In addition, a cluster of GO terms related to oxidoreductase activity, monooxygenase activity, peroxidase activity, protein serine kinase activity, naringenin-chalcone synthase activity, protein serine/threonine kinase activity, hormone binding, glucosidase activity, and phenylalanine ammonia-lyase activity were also observed in the molecular function category (Figure 2, Supplementary Table S1). Thus, upregulated DEGs were assigned to GO terms mainly associated with defending the soybean plant in response to *C. cassiicola* infection.

**Figure 3:**
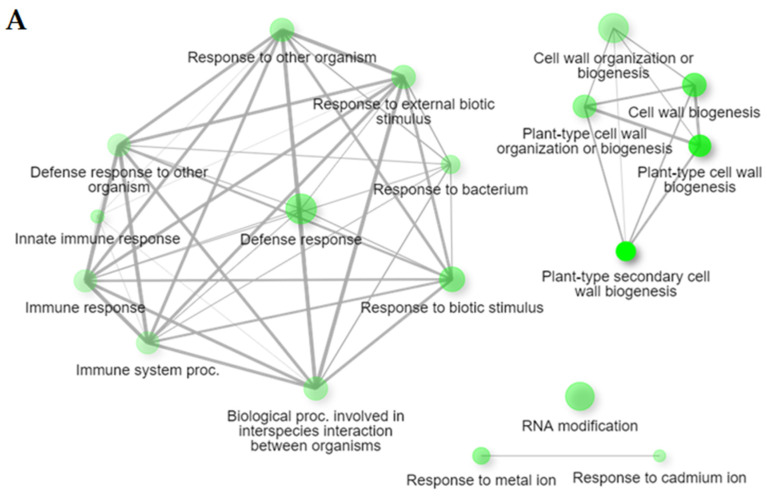

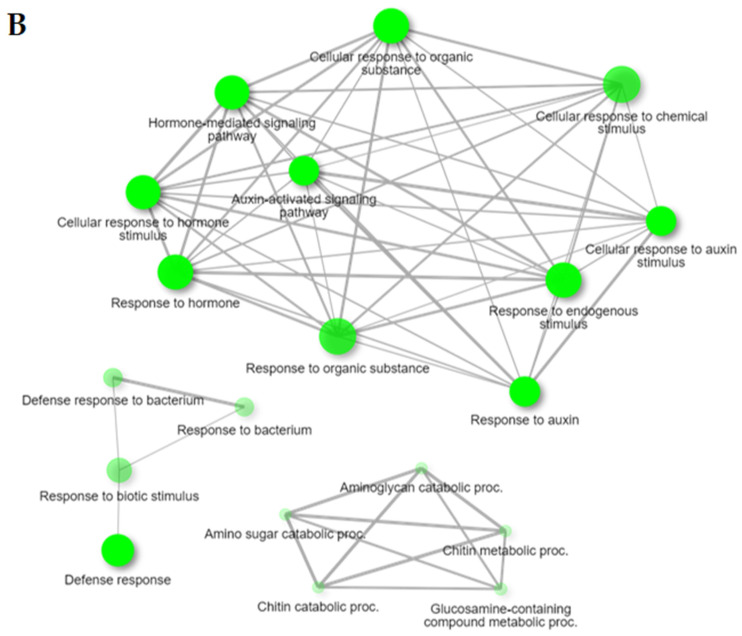
The interactive plot displays the relationship between significant enriched biological processes. Activation of enrichment network of upregulated genes in resistant genotypes in response to *C. cassiicola* infection but not observed in susceptible (**A**) Bedford (**B**) Council. The circles on the plots represent nodes (different biological processes), and lines represent a connection between two biological processes.

The GO analysis (based on uniquely identified DEGs for each genotype) identified in Bedford were: gene functions involved in biotic stimulus, the defense response to other organisms, immune system process, immune response, defense response, innate immune response, plant type secondary cell wall biogenesis, response to external biotic stimulus, cell wall organization and biogenesis (Figure 3A, Supplementary Table S2). In Council, unique upregulated DEGs have enriched biological processes, GO terms involved in the defense response, the chitin metabolic process, chitin catabolic process, hormone-mediated signaling pathway, response to auxin, response to biotic stimulus, induced systemic resistance, activation of an innate immune response, and the auxin-activated signaling pathway. Protein serine/threonine kinase activity, chitin binding, chitinase activity, and oxidoreductase activity were upregulated in molecular functions after infection compared to susceptible genotypes. Moreover, a cluster of GO terms related to meiotic nuclear division, the meiotic cell cycle process, chromatin assembly, nucleosome assembly, chromosome segregation, chromosome organization, and nuclear division was downregulated after *C*. *cassiicola* infection in Council, which indicates that cell division processes might be affected (Figure 3B, Supplementary Table S2).

In susceptible genotypes, the enriched GO terms assigned to unique DEGs were not involved directly with the defense network. The most enriched upregulated biological process GO terms in Henderson were rRNA processing, the RNA metabolic process, and ribosome biogenesis. Likewise, in Pembina, RNA modification, the phenylpropanoid metabolic process, and the lignin metabolic process were the most enriched upregulated GO terms in the biological process (Supplementary Table S2).

### 2.3. KEGG Pathway Analysis

The KEGG enrichment analysis utilized the DEGs shared by all four genotypes at 24 hpi or 48 hpi against the *Glycine max* gene background, assigned to 98 pathways; 18 pathways were significantly enriched (FDR—adjusted *p* ≤ 0.05) [31]. The metabolic pathways (gmx01100), biosynthesis of secondary metabolites (gmx01110), phenylpropanoid biosynthesis (gmx00940), plant hormone signal transduction (gmx04075), starch and sucrose metabolism (gmx00500), MAPK signaling pathway (gmx04016), circadian rhythm (gmx04712), and flavonoid biosynthesis (gmx00941) were prominent pathways in response to *C. cassiicola* at 24 and 48 hpi across all genotypes. In addition, isoflavonoid biosynthesis, cutin, suberine and wax biosynthesis, fatty acid elongation, glycerolipid metabolism, ascorbate and aldarate metabolism, cyanoamino acid metabolism, thiamine metabolism, carotenoid biosynthesis, nitrogen metabolism, ubiquinone, and other terpenoid-quinone biosynthesis were also enriched (Table 2).

**Figure 4:**
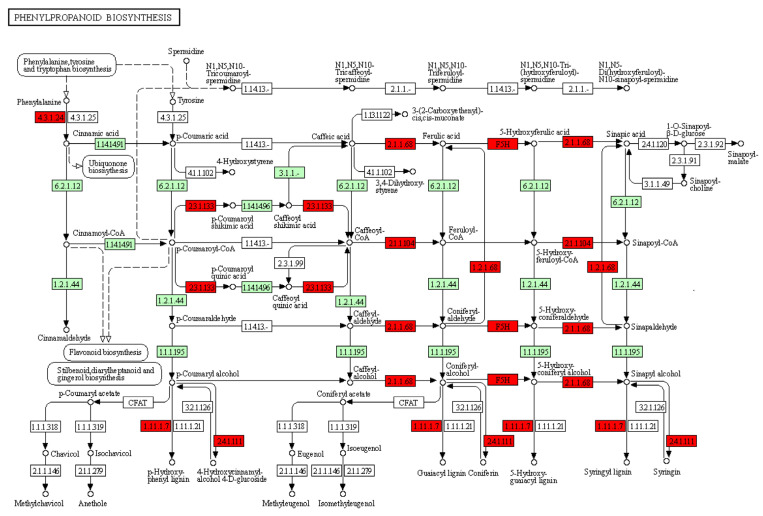
Illustration of the phenylpropanoid biosynthesis pathway. The numbers in the boxes represent coding for enzymes. The red-colored boxes represent upregulated genes in all four genotypes after infection. The green boxes in the pathway diagram represent remaining genes or enzymes derived from the soybean genome and are involved in the pathway under investigation.

A closer investigation of the phenylpropanoid pathway revealed that many DEGs are involved in the biogenesis of various phenolic and lignin compounds. Figure 4 illustrates the activation of various phenolic polymers and lignin compounds in the phenylpropanoid pathway. The upregulated DEGs were phenylalanine ammonia-lyase (EC:4.3.1.24), caffeate O-methyltransferase (EC 2.1.1.68), coniferyl-aldehyde dehydrogenase (EC:1.2.1.68), shikimate O-hydroxycinnamoyltransferase (EC:2.3.1.133), coniferyl-alcohol glucosyltransferase (EC:2.4.1.111), and 1-cys peroxiredoxin (EC:1.11.1.7). This indicates that these pathways may increase soybean immunity against *C. cassiicola* (Figure 4).

Several upregulated DEGs were assigned to the biosynthesis of the flavonoid/isoflavonoid pathway: IFS1 (isoflavone synthase 1 precursor), IOMT1 (isoflavone 4′-O-methyltransferase), isoflavone 7-O-methyltransferase (EC:2.1.1.150), isoflavone 7-O-glucoside-6″-O-malonyltransferase (EC:2.3.1.115), isoflavone/4′-methoxyisoflavone 2′-hydroxylase (EC:1.14.14.90; 1.14.14.89), and CYP93A1 (3,9-dihydroxypterocarpan 6A-monooxygenase). Similarly, in the flavonoid biosynthesis pathway, upregulated DEGs were CHS8 (chalcone synthase), shikimate O-hydroxycinnamoyltransferase (EC:2.3.1.133), caffeoyl-CoA O-methyltransferase (EC:2.1.1.104), flavonoid 3′-monooxygenase (EC:1.14.14.82), flavonoid 4′-O-methyltransferase (EC:2.1.1.231), and flavanone 4-reductase (EC:1.1.1.219; 1.1.1.234) (Supplementary Figure S1A,B).

### 2.4. Identification of Differentially Expressed Transcription Factors (TFs)

**Figure 5:**
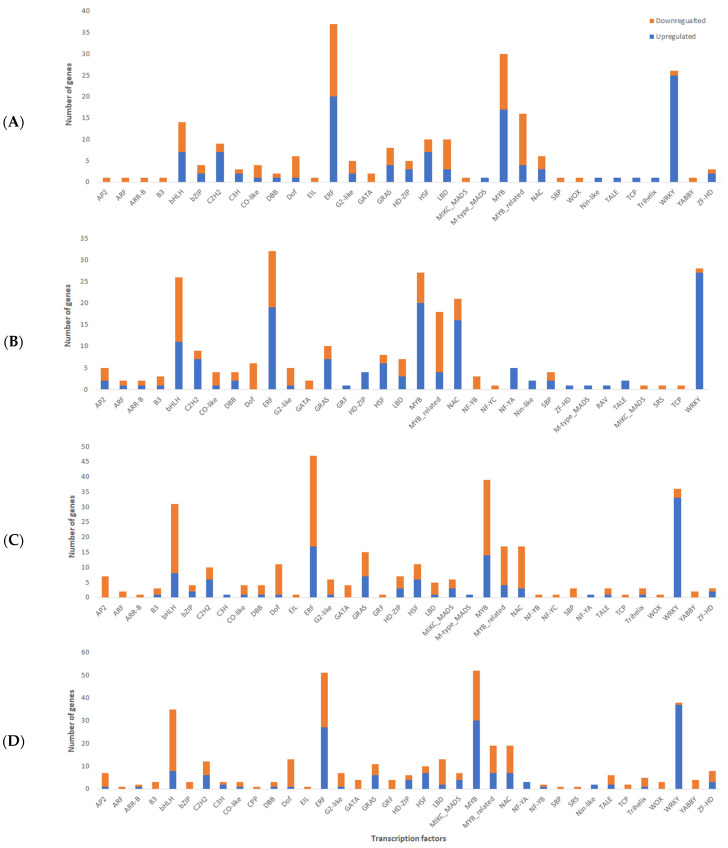
Classification of transcription factor families for four genotypes: (**A**) Council, (**B**) Bedford, (**C**) Henderson, and (**D**) Pembina.

For transcription factors (TF), 574 DEGs were identified in 40 different TF families across all four genotypes. The highest represented TF families are 80 DEGs in ethylene responsive factor (ERFs), 74 MYB, 51 WRKY, 62 bHLH, and 38 NAC. Our study found that WRKY TFs were predominantly upregulated after infection, suggesting that this particular group might have a critical role in response to *C. cassiicola* (Figure 5). Furthermore, a total of 16 DEGs belonging to the WRKY family were upregulated in all four genotypes after *C. cassiicola* infection (Figure 5).

In Council, 45 DEGs belonging to 22 TFs families were observed with upregulated TFs distributed between the C2H2 family (3 TFs), WRKY (1 TFs), NAC (1 TFs), MYB (2 TFs), and MYB-related family (1 TFs). In Bedford, 66 genes belonging to 24 TF families were differentially expressed after *C. cassiicola* infection. Among them, upregulated TFs were distributed in NAC (9 TFs), WRKY (4 TFs), bHLH (4 TFs), ERF (3 TFs), MYB (3 TFs), and MYB-related family (2 TFs). These upregulated TFs may enhance resistance in genotypes against *C. cassiicola* infection. Interestingly, there was no expression change in these TFs in susceptible genotypes after *C. cassiicola* infection.

### 2.5. Quantitative Real-Time Expression Analysis

**Figure 6:**
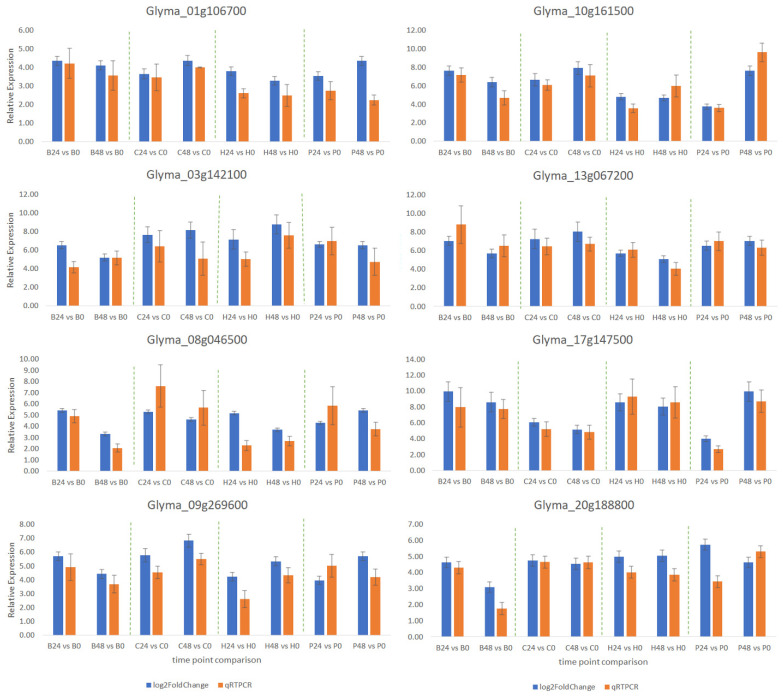
Quantitative real-time PCR (qRT-PCR)-based validation of DEGs in response to *C. cassiicola* inoculation at different time points. Relative gene expression is represented in Log2fold change obtained from RNA-Seq, and fold changes in qRT-PCR are calculated using the 2^−ΔΔCT^ method.

Comparing the log2 fold change from RNA-Seq analysis for eight differentially expressed genes with qPCR results revealed a positive correlation (r = 0.81) and had consistent expression trends (Figure 6). These results suggest the reliability of RNA-seq in analyzing the transcriptome of resistant and susceptible plants after *C. cassiicola* infection.

## 3. Discussion

Target spot, caused by *C. cassiicola*, is an emerging problematic disease in regions with warm and humid climates; this RNA-Seq study, involving two resistant genotypes (Bedford and Council) and two susceptible genotypes (Pembina and Henderson), sheds light on possible soybean responses to *C. cassiicola* infection and potential resistance mechanisms. The Illumina sequencing for 36 RNA-Seq libraries generating 94 GB of data was used for further analysis. An average of 25.72 million clean reads per library was obtained, of which 88.76% were mapped to the soybean genome. This indicates that our data are sufficient for conducting DEG analysis to identify defense-related genes and pathways against *C. cassiicola*.

During their life cycle, soybean plants are attacked by various pathogens (fungi, nematodes, bacteria, and viruses), which impact plant growth and development, ultimately reducing the yield [32]. Plants respond to pathogen attacks by changing their expression of genes, which alters different pathways [33]. The functional analysis of common DEGs in all four genotypes indicated that several genes belonging to biological processes, cellular components, and molecular function were influenced by *C. cassiicola* infection. Enriched GO terms of interest in all four genotypes altered in response to *C. cassiicola* infection were: defense response, response to biotic stimulus, cutin biosynthetic process, protein serine/threonine kinase, oxidoreductase activity, and peroxidase activity. These pathways have been highlighted in several plant–pathogen interaction transcriptomic studies such as *Athelia* (*Sclerotium*) *rolfsii* in peanut [34] and *Xanthomonas* sp. in pepper [35] and tomato [36]. Moreover, upregulated genes only in Bedford and Council after *C. cassiicola* infection were assigned to the defense response, response to biotic stimulus, and immune response. Additionally, upregulated genes found only in Council (the genotype with the highest level of target spot resistance) exhibit the activation of chitin-binding/catabolic, chitinase activity, the salicylic acid biosynthetic process, hormone-mediated signaling pathway, and protein serine/threonine kinase activity. These unique upregulated DEGs found in the resistant genotypes after infection might contribute to resistance to *C. cassiicola*. Transcriptome studies involving *C. cassiicola* infection of rubber found similar activation of the defense response and chitinase activity only in a tolerant clone [27,28].

Fungal invasion of plants triggers two layers of immune defense mechanisms. Transmembrane proteins such as receptor kinases (RLKs) and receptor-like proteins (RLPs) act as pattern-recognition receptors (PRRs), known as host sensors, that allow plants to recognize microbial pathogens, surrounding them through pathogen-associated molecular pattern (PAMP)-triggered immunity (PTI) [37]. This PTI is the first line of defense that restricts pathogen invasion. Effector-triggered immunity (ETI) is the second layer of defense against pathogen attack. This system recognizes the effector proteins that cause local cell death, often called the hypersensitive reaction (HR) in plants [33]. A leucine-rich repeat (LRR) domain is generally present in immune receptors and defense genes of plants. Several genes with the LRR domain were upregulated in all four genotypes after *C. cassiicola* infection. Specifically, 73 and 33 LRR genes were upregulated in Council and Bedford, while no expression difference was found in susceptible genotypes. These genes found in Council and Bedford were in the disease resistance protein (TIR-NBS-LRR class) family (10, 6), Leucine-rich receptor-like protein kinase family (10, 2), LRR family protein (22, 14), LRR protein kinase family (19, 8), LRR transmembrane protein kinase (10, 3), LRR and NB-ARC domains containing disease resistance protein (1, 0) and the MLLR family (1, 0), respectively.

The most well-known disease-resistant genes (R genes) contain a nucleotide-binding site (NSB) and LRR protein, which helps in the identification of specialized pathogen-associated proteins [38]. These genes can be further classified into proteins with the north terminal toll and interleukin 1 receptor (TIR) domain, coiled-coil (CC) domain, and without any N domain [39]. Interestingly, it was observed that all additional upregulated NBS-LLR genes in Council and Bedford had a TIR domain. Two different TIR-NB-LLRs were identified in *Arabidopsis*, providing tolerance to *Leptosphaeria maculans* fungus [40]. Another gene, *RLM3*, encoding the TIR-NB class, was found to provide immunity to different necrotrophic fungal pathogens in *Arabidopsis* [41]. Further investigation is needed to understand the role of TIR-NBS-LLR genes in the soybean defense response to *C. cassiicola*.

**Figure 7:**
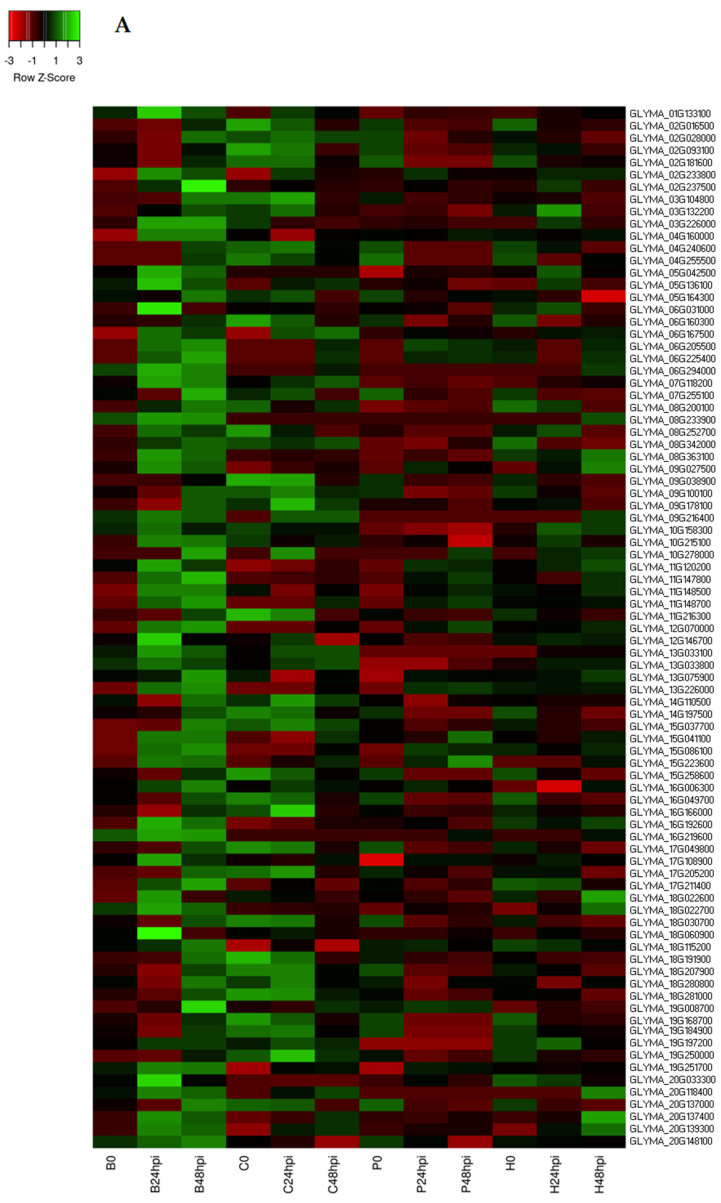

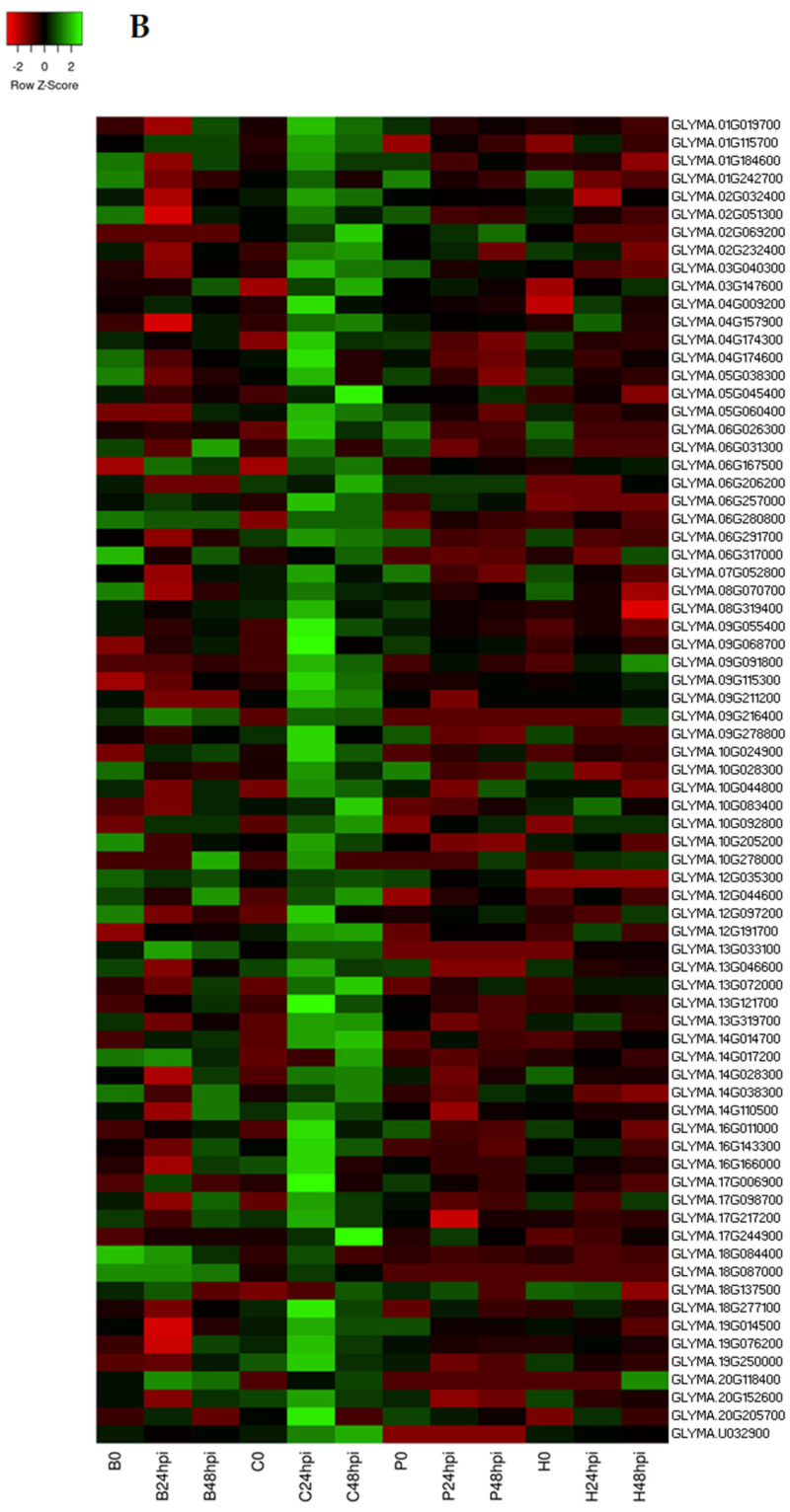
Heat maps represent the log2fold-change-based expression pattern of defense-related genes in (**A**) Bedford and (**B**) Council after *C. cassiicola* inoculation compared to susceptible genotypes at different time points. The annotation of these genes was retrieved from Soybase.

Receptor kinases send downstream signals for an appropriate cellular response to biotic and abiotic stress. Some receptor kinases contain cysteine-rich proteins, known as cysteine-rich receptor-like kinases (CRKs). Genes in the CRK family play important roles in disease resistance by interacting with PAMP and sending defense signaling for an HR-like cell death [42,43,44]. A total of 46 and 42 CRKs genes were upregulated in Council and Bedford, respectively, after *C. cassiicola* infection. Interestingly, two copies of the CRK 25 gene (Glyma.20g137400 and Glyma.20g139300) were highly upregulated in Bedford and a copy of CRK 4 (Glyma.20g118400) was upregulated in both resistant genotypes but had very low expression at 24 hpi in both susceptible genotypes (Figure 7A,B). CRK 4 is an important receptor protein kinase identified to have a critical role in early PTI response by triggering HR [42,43,44]. This suggests that early activation of CRK genes in resistance genotypes is vital to reducing the colonization of *C. cassiicola*.

Chitin is a cell wall component of fungi that is not present in the plant cell wall [45]. Chitinase possesses antifungal properties, restricting the growth of many fungal pathogens such as *Trichosanthes dioica*, *Aspergillus niger*, *Alternaria solani*, *Fusarium* spp., *Rhizoctonia solani*, and *Verticillium dahlia* [46,47,48,49]. Chitinase A (Glyma.19G076200) was highly expressed in Council when compared to susceptible genotypes at 24hpi and 48 hpi (Figure 7B). Tobacco plants overexpressing Chitinase A from *Autographa californica* nuclear polyhedrosis virus showed resistance to fungal pathogens [50]. Thus, higher expression of Chitinase A might reduce in planta colonization of *C. cassiicola*.

Other defense-related genes were upregulated in resistant genotypes with significantly higher expression than susceptible genotypes at 24 and 48 hpi. In Council, these genes belong to disease-resistance-responsive dirigent-like protein (Glyma.03g147600), B-box zinc finger (Glyma.04g009200), mitogen-activated protein kinase (Glyma.12g097200), cysteine-rich secretory protein (Glyma.16g143300, *NPR1*), NB-ARC domain-containing disease resistance protein (Glyma.18g084400, *RPM1*) and Glyma.18g087000, *RPM4*), receptor serine/threonine kinase (Glyma.13g033100), and serine protease inhibitor (Glyma.20g205700) (Figure 7B). The *RPM1* gene provides resistance in *A. thaliana* against *P. syringae* [51] and in wheat against *Puccinia striiformis* [52]. *RPM* gene families have been identified as major players in the soybean defense mechanism against pathogens and in the stress response [53,54]. Pathogenesis-related proteins (PR) in plants participate in the innate immune system defense response against pathogens. Several studies have found that the *NPR1* gene provides resistance to different species of fungus in cotton [55], *Arabidopsis* [56], and *Brassica juncea* [57]. In Bedford, these genes belong to wall-associated kinase family protein (Glyma.09g027500), mitogen-activated protein kinase (Glyma.18g060900, *MAPK*), *kunitz trypsin inhibitor 1* (Glyma.08g342000, *KTI1*), pentatricopeptide repeat (PPR) superfamily protein (Glyma.08g233900), scorpion toxin-like knottin superfamily protein (Glyma.06g160300), cytochrome P450 (Glyma.07g118200 and Glyma.05g042500), receptor serine/threonine kinase (Glyma.13g033100 and Glyma.13g033800), and peroxidase superfamily protein (Glyma.02g233800) (Figure 7A). The transcriptomic study revealed the expression of some DEGs associated with MAPK cascades in response to *Xanthomonas oryzae* infection in a resistant rice genotype [58]. Moreover, the overexpression of KTI1 in tobacco enhances the resistance to *Rhizoctonia solani* infection [59]. There is a need to understand the impact of these upregulated genes relative to target spot resistance.

In this study, *C. cassiicola* infection was associated with diverse plant defense response TF families, such as ERFs, WRKY, MYB, and bHLH. For the WRKY TF family, WRKY29 (Glyma.08G018300) in Council, and WRKY6 (Glyma.08G320200, Glyma.18G092200), WRKY7 (Glyma.17G239200), and WRKY41 (Glyma.19G254800) in Bedford were upregulated after *C. cassiicola* infection, while in the susceptible genotypes, there was no expression difference between control and infected tissue. Previous studies show that the expression of WRKY29 and WRKY41 TFs increased Fusarium head blight resistance in wheat [60] and *Pseudomonas* resistance in Arabidopsis [61].

Phenylpropanoid biosynthesis plays an essential role in the plant stress response. This pathway leads to the biogenesis of various phenolic polymers, lignin compounds, and flavonoids, increasing plant immunity [62,63]. The gene coding for phenylalanine ammonia-lyase (PAL), caffeate O-methyltransferase (CCoAOMT), coniferyl-aldehyde dehydrogenase, 1-cys peroxiredoxin, shikimate O-hydroxycinnamoyltransferase, and coniferyl-alcohol glucosyltransferase was found to be upregulated in all four genotypes. This indicates that this pathway might be stimulated early in all genotypes as part of the PTI response. Similar results were observed in the research conducted on rubber tree clones in response to *C. cassiicola* infection [27]. Moreover, these gene-coding enzymes form syringyl and guaiacyl, units of lignin polymers, which are major building blocks of lignin and end products of lignin biosynthesis. Phenylpropanoid polymer lignin acts as a physical barrier against pathogen invasion [64]. The phenylpropanoid pathway is regulated in stress conditions and associated with the lignification process in *Arabidopsis* and *Populous* [65]. Similarly, lignin formation is an essential process for the defense of host plants under both abiotic and biotic stresses [66,67].

**Figure 8:**
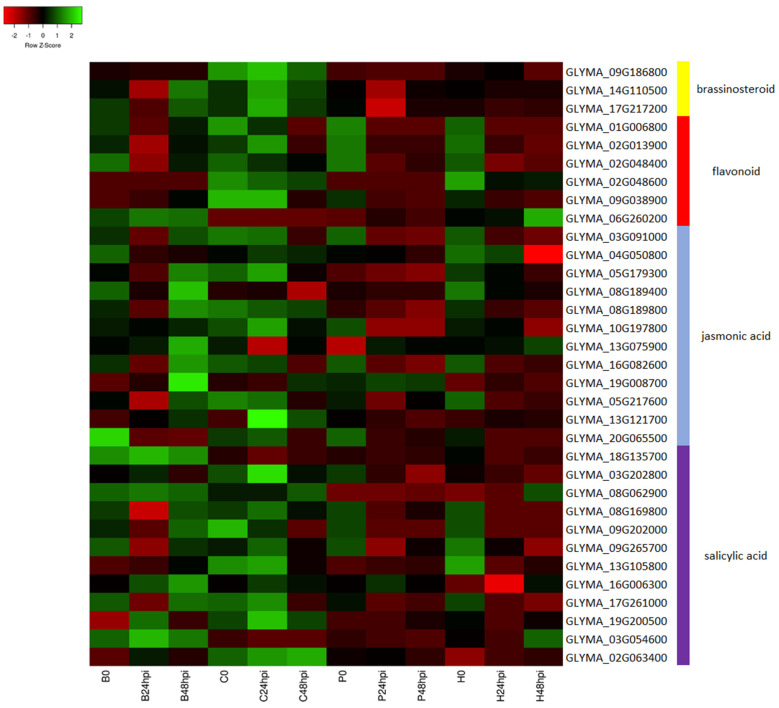
The heat map represents the log2fold-change-based expression pattern of secondary metabolites biosynthesis DEGs in Bedford and Council after *C. cassiicola* inoculation at different time points compared to susceptible genotypes. The annotation of these genes was retrieved from Soybase.

Flavonoid biosynthesis is an essential downstream branch of phenylpropanoid metabolism. The gene expression of chalcone synthase, the key enzyme in the flavonoid pathway, is induced in plants with fungal or bacterial infection [68]. Similarly, isoflavonoids are a mainly legume-specific subclass of flavonoid metabolites with significant roles in plant defense [69]. In the enriched isoflavonoid pathway, three cytochrome families were involved in the biosynthesis process: CYP93C (cytochrome P45093C), CYP81E1/E7, (isoflavone/4′-methoxyisoflavone 2′-hydroxylase) and CYP93A1 (3,9-dihydroxypterocarpan 6A-monooxygenase). These pathways were activated in both resistant and susceptible genotypes in this study, suggesting a common defense pathway activated in soybean plants when attacked by *C. cassiicola*. Additionally, few genes in the flavonoid biosynthesis pathway were more highly expressed in Council and/or Bedford (Figure 8). In Bedford, two genes, Glyma.09g038900 (*MYB111*) and Glyma.06g260200 (NAD(P)-linked oxidoreductase), involved in flavonoid biosynthesis were upregulated and more highly expressed than in the susceptible genotypes after infection. Similarly, in Council, four genes involved in flavonoid biosynthesis had higher expression than in susceptible genotypes after infection: Glyma.01g006800 (pectin lyase-like superfamily protein), Glyma.02g013900 (MYB domain protein 12), Glyma.02g048400 (flavanone 3-hydroxylase), and Glyma.02g048600 (flavanone 3-hydroxylase). MYB transcription factors such as *MYB12* and *MYB111* modulate the production flavonoid pathway by regulating early biosynthesis genes such as chalcone synthase (*CHS*), chalcone isomerase (*CHI*), flavanone 3-hydroxylase (*F3H*), and flavonol synthase1 (*FLS1*) during normal development stages and in stress conditions [70,71,72,73]. Two different copies of flavanone 3-hydroxylase (*F3H*) were more highly expressed in Council compared to susceptible genotypes at 24 hpi and 48 hpi. Flavanone 3-hydroxylase (*F3H*) is responsible for producing different flavonoid compounds. Studies have associated the upregulation of genes with tolerance/resistance to pathogens such as *Alternaria solani*, *Ascochyta rabiei (Pass) Labr.*, and Xanthomonas oryzae pv. oryzae [74,75,76]. Thus, our study speculates a major role of genes involved in the flavonoid pathway in contributing to resistance to *C. cassiicola*.

Jasmonic acid (JA), salicylic acid (SA), and brassinosteroids (BRs) are the plant defense hormones activated in the downstream process of PTI and ETI responses [77,78]. In this study, genes associated with the biosynthesis of BRs, JA, and SA were upregulated in resistant genotypes compared to susceptible genotypes after *C. cassiicola* infection. Three and two genes related to BR biosynthesis were highly expressed after infection in Council and Bedford, respectively (Figure 8). Furthermore, twelve genes for each SA and JA were upregulated in at least one resistant genotype while having lower expression in susceptible genotypes after infection. Such higher expression of genes involved in these defense-related hormones might play a vital role in conferring target spot resistance. Similar results were observed in other transcriptomic studies involving plant host–pathogen interactions [34,79,80].

Quantitative disease resistance (QDR) is a phenomenon wherein many genes with small effects are differentially expressed during the invasion of the pathogen, which results in a reduction in fungal colonization. Pyramiding such small effect genes from the different resistant genotypes would be an effective strategy to develop an enduring disease-resistant variety [81,82]. Studies have demonstrated a higher level of disease resistance by pyramiding such QDRs [83,84]. Further research needs to be conducted to understand the effects of QDRs and genetic gain for disease resistance by pyramiding these QDRs from Bedford and Council into a single germplasm. This would also help to develop germplasm with broad-spectrum resistance as QDR generally participates in defense mechanisms against a wide range of microbial pathogens and multiple races [82,85].

## 4. Materials and Methods

### 4.1. Plant Materials and Inoculation

The experiment was performed using two resistant soybean genotypes, ‘Council (PI 587091)’ and ‘Bedford (PI 548974)’, and two susceptible genotypes, ‘Henderson (PI 665225)’ and ‘Pembina (PI 638510)’, which were selected based on a screening study conducted [9]. In the greenhouse, soybean seeds were sown in twelve separate 11.5 × 11.5 cm^2^ pots filled with PRO-MIX BX (Premier Tech Horticulture, Quakertown, PA, USA). Plants were watered as needed under a photoperiod of 14/10 (light/dark) and a thermocycle of 24 °C/12 °C for 25 days (growth stage V3–V4) before fungal infection. An isolate of *C. cassiicola* (LIM01) was grown on V8 agar plates for twelve days at 28 °C with 12/12 h of light/dark in an incubator and then conidia were scraped into sterile distilled water and filtered with cheesecloth to make a conidial suspension with a concentration of 50,000 per mL [9]. Plants were sprayed with 0.05% of a Tween 20 solution and allowed to dry for 5 min before inoculation. The freshly prepared conidial suspension was sprayed onto the axial and abaxial leaf sides using a fine mist professional spray bottle (Spray Pro) until run-off. Afterward, inoculated plants were transferred into a plastic mist chamber inside the greenhouse with a non-inoculated control of each genotype (sprayed with distilled water). Plants were arranged in a completely randomized design (CRD) in the mist chamber; the mist ran for 2 s every 10 min for three days to maintain high humidity [9].

### 4.2. RNA Extraction, Library Preparation, and Illumina Sequencing

Three biological replicates from all four genotypes at 24 and 48 h post-inoculation (hpi), and their respective controls, were selected for RNA isolation. RNA was extracted from 100–150 mg tissue samples using the Direct-zol RNA Mini-Prep Kit (Zymo Research, Irvine, CA, USA) and the concentration was estimated using a Nanodrop 2000 spectrophotometer (ThermoFisher Sci., Waltham, MA, USA). The RNA degradation was evaluated using agarose gel electrophoresis and intact RNA was sent for RNA sequencing (RNA-Seq). Novo gene Bioinformatics Technology Co., Ltd., Sacramento, CA, USA, performed cDNA library construction of 36 samples (4 genotypes × 3 treatments × 3 replicates) and 150 bp paired-end sequencing run on an Illumina NovaSeq 6000 (Illumina Inc., San Diego, CA, USA). Data were released after a read quality check for a percentage of reads containing N > 10% (N represents the base that cannot be determined) and low-quality reads (Q score ≤ 5).

### 4.3. RNA-Seq Data Analysis

An additional quality check of raw reads was performed using fastp software v0.23.1 [86] to remove adapters, Poly A sequences, low-quality reads (Q < 30), and reads < 15 bp in length after trimming. These cleaned reads were mapped to the soybean (*Glycine max* (L.) Merr.) reference genome [13], and transcript quantification was performed using Salmon ver.0.9.1 [87]. A total of eight comparisons, which include 24 hpi vs. control and 48 hpi vs. control for all four genotypes, were performed to identify differentially expressed genes (DEGs) using R-package DESeq2 (Version 1.37.6) [88]. The genes with an absolute value of log2 fold change ≥2 (upregulated genes) or ≤−2 (downregulated genes) and a false discovery rate (FDR) < 0.01 were considered significant DEGs.

### 4.4. GO Enrichment and KEGG Pathway Analysis

Gene ontology functional enrichment (GO) analysis for the DEGs was conducted using ShinyGo v 0.76 [89] with *Glycine max* as the background. Gene ontology provided annotations at cellular, molecular, and biological levels. The function categories of enriched GO terms were considered significant with an FDR-adjusted *p* ≤ 0.05.

KEGG pathway analysis of DEGs was carried out using KOBAS v 3.0 software [31] with *Glycine max* sequences as the background, a hypergeometric test, and Bonferroni FDR correction (FDR *p* ≤ 0.05). This analysis tests the statistical enrichment of DEGs in KEGG pathways.

### 4.5. Identification of Transcription Factors (TFs)

PlantTFDB (http://planttfdb.gao-lab.org/ (accessed on 10 January 2022)) was used to identify transcription factors involved in regulating the soybean response after *C. cassiicola* infection, containing 58 plant transcription factor (TF) families from 165 plants [90]. The analysis was conducted using the TF enrichment tool and soybean (*G. max*) transcription factor database, which contains 6150 TFs (3747 loci) distributed into 57 families. TFs were searched in differentially expressed genes at 24 hpi or 48 hpi for each genotype.

### 4.6. Quantitative Real-Time PCR Validation

Quantitative real-time PCR (qRT-PCR) was performed for the relative expression of selected eight DEGs to validate RNA-Seq data. All of the eight randomly selected gene sequences were retrieved from Soybase (https://soybase.org/ (accessed on 5 July 2022)). RNA extraction was conducted as described above, and cDNA synthesis was carried out using a qScript™ cDNA Synthesis Kit (New England Biolabs, Inc., Ipswich, MA, USA). Primers were designed using the Primer-BLAST tool [91] and listed in Supplementary Table S3. The qRT-PCR was performed with the Thermo Fisher Scientific Biosystems StepOnePlus™ Real-Time PCR system (Applied Biosystem, MA, USA) using PerfeCTa SYBR Green ROX FastMix (Quantabio). The following conditions were used for amplification: 2 min at 95 °C followed by 40 cycles of 5 s at 95 °C and 10 s at 58 °C plus melting curves to verify PCR products. The gene expression level of selected genes was calculated with the 2^−ΔΔCT^ method [92], and the ubiquitin-conjugating protein endogenous control was used to normalize the variance among samples.

## 5. Conclusions

This study presents the first large-scale comparative transcriptomic profiling of resistant and susceptible soybean genotypes in response to the invasion of the necrotrophic fungus *C. cassiicola*. The results revealed a complex and massive gene network response, providing insight into mechanisms directing resistance to *C. cassiicola* in soybean. The analysis suggests that the TIR-NBS-LRR, LRR, NB-ARC, CRKLs, and DIR genes play an essential role in understanding pathogen invasion through a downstream resistance mechanism. Furthermore, genes involved in flavonoid/isoflavonoid, phenylpropanoid, JA, SA, and BA are upregulated upon *C. cassiicola* attack, thereby inducing systemic resistance. 

## Figures and Tables

**Table 1:** An overview of statistics for the quality of sequencing data.

Samples	Raw Reads	Clean Reads	Map Reads	Raw Data (G)	Clean Data (G)	Q30 (%)	GC (%)
Bedford—0 hpi	26,390,793	25,793,638	23,582,978	7.9	7.8	93.39	45.04
Council—0 hpi	23,089,787	22,563,156	20,640,957	6.9	6.8	93.53	45.19
Henderson—0 hpi	22,838,228	22,381,404	20,209,026	6.9	6.7	93.19	44.79
Pembina—0 hpi	25,554,633	25,099,735	22,617,422	7.7	7.5	93.39	44.72
Bedford—24 hpi	28,423,021	28,174,656	24,664,671	8.6	8.5	93.19	44.69
Council—24 hpi	26,514,264	26,147,721	23,551,179	8.0	7.8	93.17	45.18
Henderson—24 hpi	27,493,410	27,128,017	23,310,773	8.2	8.1	93.05	44.69
Pembina—24 hpi	33,485,280	33,067,807	29,065,093	10.1	9.9	93.18	44.42
Bedford—48 hpi	25,860,451	25,641,661	22,746,284	7.8	7.7	93.37	44.94
Council—48 hpi	23,417,306	23,237,271	20,529,754	7.0	7.0	93.31	45.03
Henderson—48 hpi	25,075,023	24,887,851	21,578,946	7.5	7.4	93.09	44.53
Pembina—48 hpi	24,925,859	24,478,453	21,424,984	7.5	7.3	93.26	44.54

**Table 2:** Significantly enriched KEGG pathways of differentially expressed genes (DEGs) common in four genotypes.

Term	ID	Input Number	Background Number	*p*-Value	Corrected *p*-Value
Biosynthesis of secondary metabolites	gmx01110	129	2121	1.83 × 10^−19^	1.79 × 10^−17^
Metabolic pathways	gmx01100	180	4144	6.06 × 10^−13^	2.97 × 10^−11^
Phenylpropanoid biosynthesis	gmx00940	35	349	2.87 × 10^−11^	9.37 × 10^−10^
Circadian rhythm—plant	gmx04712	18	106	1.29 × 10^−9^	3.17 × 10^−8^
Cyanoamino acid metabolism	gmx00460	15	87	2.53 × 10^−8^	4.97 × 10^−7^
Flavonoid biosynthesis	gmx00941	15	94	6.30 × 10^−8^	1.03 × 10^−6^
Isoflavonoid biosynthesis	gmx00943	10	42	4.13 × 10^−7^	5.78 × 10^−6^
Starch and sucrose metabolism	gmx00500	23	299	4.73 × 10^−6^	5.80 × 10^−5^
Thiamine metabolism	gmx00730	7	43	0.000191	0.00199
Ubiquinone and other terpenoid-quinone biosynthesis	gmx00130	10	93	0.000203	0.00199
Fatty acid elongation	gmx00062	7	49	0.000391	0.003485
MAPK signaling pathway—plant	gmx04016	19	307	0.00043	0.003513
Plant hormone signal transduction	gmx04075	30	675	0.00233	0.017564
Ascorbate and aldarate metabolism	gmx00053	8	90	0.002635	0.018442
Cutin, suberine, and wax biosynthesis	gmx00073	6	54	0.003245	0.021197
Nitrogen metabolism	gmx00910	6	65	0.007429	0.043641
Carotenoid biosynthesis	gmx00906	6	66	0.007942	0.043641
Glycerolipid metabolism	gmx00561	10	158	0.008016	0.043641

## Data Availability

The data presented in this study are available on request to the corresponding author.

## Supplementary Materials

The following supporting information can be downloaded at: https://www.mdpi.com/article/10.3390/ijms241310563/s1.
